# Ultrastrong and Highly Sensitive Fiber Microactuators Constructed by Force‐Reeled Silks

**DOI:** 10.1002/advs.201902743

**Published:** 2020-01-16

**Authors:** Shihui Lin, Zhen Wang, Xinyan Chen, Jing Ren, Shengjie Ling

**Affiliations:** ^1^ School of Physical Science and Technology ShanghaiTech University 393 Middle Huaxia Road Shanghai 201210 China

**Keywords:** actuators, artificial muscle, force reeling, mechanical property, silk fibers

## Abstract

Fiber microactuators are interesting in wide variety of emerging fields, including artificial muscles, biosensors, and wearable devices. In the present study, a robust, fast‐responsive, and humidity‐induced silk fiber microactuator is developed by integrating force‐reeling and yarn‐spinning techniques. The shape gradient, together with hierarchical rough surface, allows these silk fiber microactuators to respond rapidly to humidity. The silk fiber microactuator can reach maximum rotation speed of 6179.3° s^−1^ in 4.8 s. Such a response speed (1030 rotations per minute) is comparable with the most advanced microactuators. Moreover, this microactuator generates 2.1 W kg^−1^ of average actuation power, which is twice higher than fiber actuators constructed by cocoon silks. The actuating powers of silk fiber microactuators can be precisely programmed by controlling the number of fibers used. Lastly, theory predicts the observed performance merits of silk fiber microactuators toward inspiring the rational design of water‐induced microactuators.

## Graphical abstract

A new kind of robust and fast‐responsive silk microactuators, by integrating experimental and theoretical designs, are presented. These microactuators feature an ultrafast response speed for humidity change with performance that can compare with the most advanced microactuator systems.

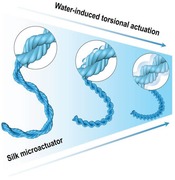

## 1 Introduction

Well‐designed fiber microactuators are widely found in nature. For example, spider dragline silk is an extraordinary fiber that surpasses the majority of conventional materials in mechanical characteristics such as the mechanical toughness. Moreover, spider dragline silk has proven to be an outstanding torsional actuator.^1^ It exhibits unique humidity‐induced actuation behavior with a torsional deformation more than 300° mm^−1^. This value is thousands of times higher than that of other conventional fiber microactuators, such as conducting polymer actuators (0.01° mm^−1^) and shape‐memory alloy fibers microactuators (0.15° mm^−1^).^1^ In fact, the humidity‐induced actuation of the spider dragline silk is even higher than that of the state of the art of the carbon nanotubes (CNTs) microactuators (250° mm^−1^),^2^, ^3^ which is powered by the electricity. This outstanding actuation characteristic promotes the spider web to maintain its geometric configuration in widely variable environmental humidity and even allows the spider to perceive the imposed external loads on the network.^4^ However, although spider silks have so many advantages for actuator applications, their practical application in the industry is not feasible yet.^5^, ^6^ Because spiders are nondomestic creatures and only produce small amounts of silk.^5^

**Figure 1:**
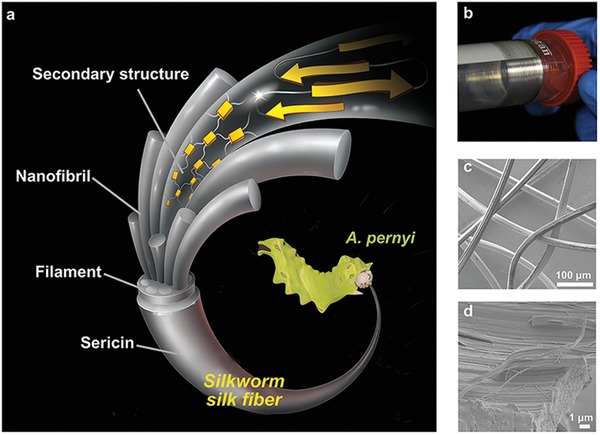
Force‐reeling of *A. pernyi* silkworm silk. a) Diagram of the hierarchical structure of the *A. pernyi* silk fiber. b) Highly lustrous FRSFs collected on centrifugal tubes. c) SEM image of FRSFs and d) SEM images of nanofibrils in the FRSF.

Silkworm silk fibers have interesting and unique structures and superior mechanical properties,^7^ as shown in **Figure** **1**. These fibers are the potential substitutions for spider silks for microactuator fabrication. However, the common‐used cocoon silk fibers (CSFs) are usually inferior to spider silks in both the structural uniformity and mechanical properties.^8^, ^9^ It may be attributed to defects and irregular changes in the structure of the natural cocoon silks, which originates from the movement of the silkworm head (Figure S1, Supporting Information). On the other hand, both the structure uniformity and the mechanical performance of silkworm silk fibers can be optimized through force‐reeling, a technique that is applied as an alternative to the natural cocooning, where the silk fiber is directly harvested from silkworm spinneret. Indeed, the silk fibers used for the structure‐and‐property investigation are often collected by the force‐reeling.^8^, ^9^, ^10^, ^11^, ^12^ However, the practical application of these force‐reeled silk fibers (FRSFs) has not achieved yet. Because there is still no highly efficient way to produce continuous and uniform fibers in large scales.^11^, ^12^

Herein, a speed‐controllable force‐reeling strategy is developed to harvest the uniform silk fiber from *Antheraea pernyi* (*A. pernyi*) silkworm with the continuous spinning length larger than 1 km. Application of FRSFs exhibits a unique trade‐off in the mechanical strength and the extensibility, resulting in the mechanical toughness almost twice higher than that of CSFs, which is even comparable with that of the spider silk. The yarn‐spinning technique can be employed to assemble FRSFs into double‐helical microactuators with the programmable actuation power, ranging from 0.77 to 2.1 W kg^−1^. This indicates the torsional power that each kilogram of the silk fiber can provide. Moreover, the detailed actuation mechanism of the FRSF microactuators is unveiled: the rough surface and the shape gradients trigger and promote the infiltration of water on the microactuator so that they can lead to the swelling and contraction of FRSFs. This synergistic effect spontaneously untwists the single FRSF yarn and maintains the overall twisting balance of the microactuator. As a pristine protein component, FRSF microactuators have broad application prospects in biomedical and smart textile fields, including the artificial muscle, biosensor, micro‐engine, and the smart textile. Furthermore, reviewing the literature confirms that silk fibers are favorable in supporting tissue formation in vitro and in modeling of time‐dependent cell responses.^13^, ^14^, ^15^

## 2 Results

### 2.1 Force‐Reeling of *A. Pernyi* Silkworm Silk

In the present study, the *A. pernyi* silkworm is selected instead of the commonly used *Bombyx mori* (*B. mori*) silkworm for the force‐reeling. This selection is done in accordance with the following interpretations. First, the primary structure of the *A. pernyi* silk is similar to that of the spider silk.^16^, ^17^ In other words, both silk proteins consist of the highly repetitive poly (alanine) and glycine‐rich domains.^18^ Second, *A. pernyi* silks are widely available, while low‐cost and sustainable.^19^, ^20^ More specifically, the global annual production of *A. pernyi* cocoons is up to 60 000 tons,^9^ and no specific feeding environment is required for *A. pernyi* silkworms.^9^, ^19^ Last but not least, in screening experiments, we found that the breeding of the *A. pernyi* silkworm was much easier than that of the *B. mori* silkworm during the force‐reeling.^9^ The *B. mori* silkworm instinctually uses facial palps to break the thread and resist the reeling force, which leads to significant fiber‐to‐fiber variability. By contrast, highly uniform FRSFs can be harvested from *A. pernyi* silkworm through continuously reeling with a speed varying from 5 to 30 mm s^−1^ for more than 8 h (see Movie S1, Supporting Information). It should be indicated that the continuous FRSFs can reach up to kilometer‐scale at the reeling speed of 20 mm s^−1^. This speed is remarkable because the obtained reeling rate is almost 2.5 times higher than the silkworm spinning speed (≈8 mm s^−1^).^21^, ^22^ The obtained fibers are highly lustrous and have uniform diameters (Figure 1b,c). Moreover, they are characterized by a strong birefringence when observed under the cross‐polarized light (Figure S2a, Supporting Information). These features are also in clear contrast with the CSFs or silk fiber spun by silkworm on a plane substrate (plane silk fiber, hereafter called the PSF). The CSF is reluster in color and has many defects on fiber surface (Figure S2b, Supporting Information).

### 2.2 Force‐Reeling of *A. Pernyi* Silkworm Silk

**Figure 2:**
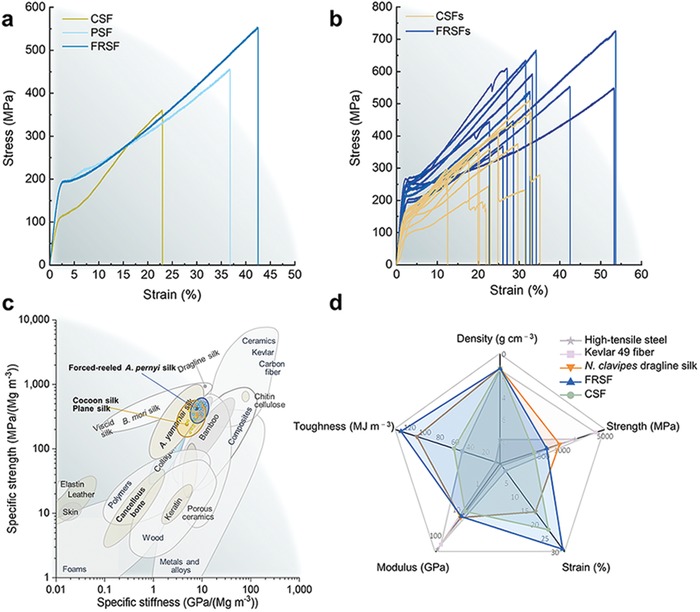
Mechanical characteristics of the FRSF. a) Typical stress–strain curves of the *A. pernyi* silk fibers spun in three different ways. b) Stress–strain curves of the CSF and the FRSF with a tensile speed of 2 mm min^−1^. The temperature and relative humidity of the test environment for the FRSF and CSF are set to 24.1 °C and 43%, and 21 °C and 42%, respectively. c) Comparison of the specific strength and the specific stiffness of the FRSF with other natural and synthetic materials. The Ashby plot was adapted from ref. 24: Adapted with permission.^24^ Copyright 2004, Taylor & Francis. d) Comparison of the density, strength, Yang's modulus, and the toughness of the FRSF with different natural and synthetic fibers. The specific values are listed in Table S1 (Supporting Information).

Mechanical characteristics are of significant importance for the practical application of an actuator. Therefore, tensile tests are initially carried out to evaluate the mechanical properties of FRSFs. Since the cross‐sections of FRSFs are not circular (Figure S3, Supporting Information), the cross‐sectional area of the single fiber instead of the diameter is utilized to calculate the corresponding tensile stress.^23^ The mechanical characteristics of the FRSFs are significantly improved and maintain the elegant trade‐off in strength, modulus, and the extensibility in comparison with those of the *A. pernyi* CSF and the *A. pernyi* PSF, respectively (**Figure** **2**a–c). For example, the strength and Young's modulus of the FRSF are 571 ± 97 MPa and 11 ± 2 GPa, respectively. These characteristics are almost 1.6 times higher than that of the CSF with strength and modulus of 359 ± 83 MPa and 7 ± 2 GPa, respectively. Furthermore, the FRSF is tougher than most of the natural and synthetic materials and is comparable with *N. clavipes* spider silk with the strain of 31 ± 6% and the toughness of 131 ± 77 MJ m^−3^.^18^, ^24^, ^25^ The trade‐off of the strength, modulus, and the toughness in the FRSF is better than those for most of the synthetic fibers (Figure 2d). For example, although the strength and the Young's modulus of Kevlar fiber approach to 3.6 and 130 GPa, the corresponding strain to failure and the toughness are only 2.7% and 50 MJ m^−3^.^26^ In other words, these characteristics are almost 14 and 3 times lower than those of the FRSF, respectively.

### 2.3 Structural Characterization

**Figure 3:**
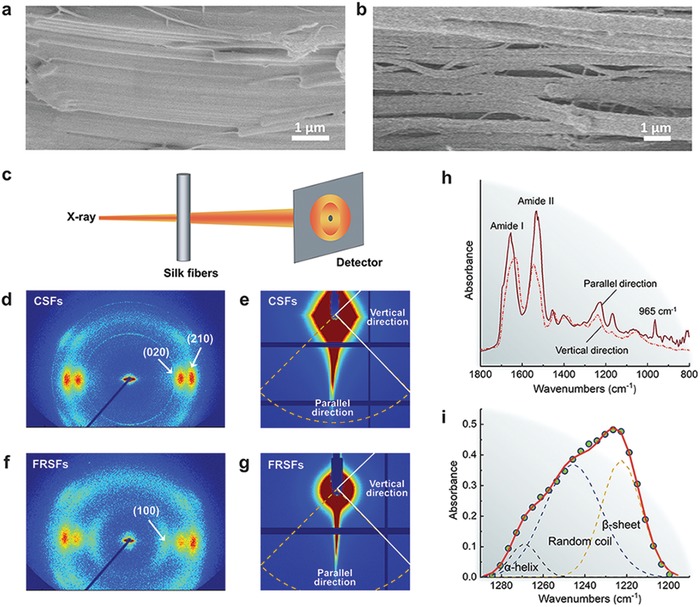
Structural characteristics of the FRSF and the CSF. a) HRSEM image of the longitudinal section of FRSF. b) HRSEM image of the longitudinal section of CSF. c) Schematic diagram of X‐ray scattering of the FRSF. d) 2D WAXS image of the CSF. e) 2D SAXS image of the CSF. f) 2D WAXS image of the FRSF. g) 2D SAXS image of the FRSF. h) S‐FTIR spectra of the single FRSF with the polarization angle of 0º and 90º. i) Deconvolution results of the amide III band of the FRSF.

High‐resolution scanning electron microscopy (HRSEM), small‐ and wide‐angle X‐ray scattering (SAXS/WAXS), and synchrotron Fourier transform infrared microspectroscopy (micro‐SFTIR) are combined to disclose the hierarchical structures of the FRSF (**Figure** **3**). Similar to animal silks, FRSFs at the mesoscale are composed of nanofibrils with the width of 5–200 nm (Figures 1d and 3a). These nanofibrils are highly oriented along the fiber axis. It is found that the orientation degree of nanofibrils in the FRSF (Figure 3g) is obviously higher than that of the CSF (Figure 3e). Moreover, precise investigation of longitudinal‐sectional HRSEM images demonstrates that the interfacial bonding and the stacking between nanofibrils in the FRSF are much stronger than those of the CSF (Figure 3b). Studies proved that the tight nanofibrils stacking is critical for improving the strength and the toughness of fibers.^27^, ^28^ This kind of self‐reinforcement is achieved by the restricted nanofibril shearing, controlled slippage, and the stress transfer.

WAXS and micro‐SFTIR results reveal the differences between the secondary structure of the FRSF and the CSF. For example, it is found that the scattering pattern of the FRSF is stronger than that of the CSF when 2θ equals to 7.23° (Figure S4, Supporting Information). The diffraction peak has been assigned to (100) reflection spacing of the β‐sheets, implying FRSFs have higher degree of crystallinity than that of CSFs (Figure S4c, Supporting Information). This finding is confirmed by the synchrotron micro‐FTIR characterization, the deconvolution of the amide III band (Figure 3i) provides an estimation of β‐sheet structure in the FRSFs of 35% to 38%, while that of the degummed *A. pernyi* CSFs are 24% to 38%.^16^, ^17^, ^29^ Therefore, it is inferred that the high modulus and tensile strength originates from the high β‐sheet contents of the FRSF. Interestingly, no significant difference in the content of β‐sheet was detected for FRSFs that were harvested from different reeling speeds. For example, the single FRSFs that were produced from reeling speeds of 5, 11, and 30 mm s^−1^ have almost the same percentage of β‐sheet, with values of 36%, 38%, and 35%, respectively (Figure S5, Supporting Information).

### 2.4 FRSF Microactuator Preparation

**Figure 4:**
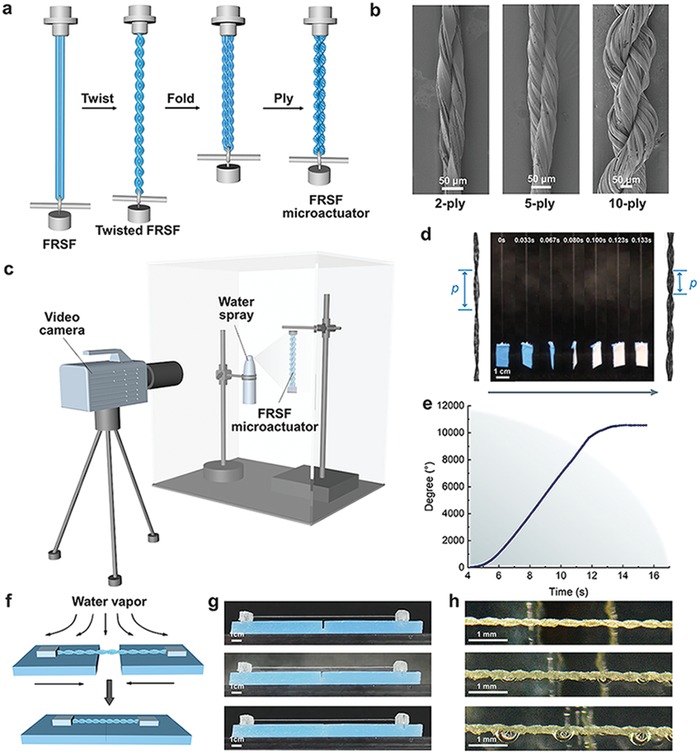
Preparation of FRSF microactuators. a) Schematic diagram of the manufacturing process of the FRSF‐based microactuator. b) SEM images of microactuators with 2, 5, and 10 ply microactuator. c) The self‐built apparatus to detect the behavior of the FRSF microactuator driven by water fog. The water fog orientation is perpendicular to the micro driver and the video camera records the status of the microactuator and the suspended paddle. d) Images of the working process of the microactuator (p is the screw pitch). e) The correlation of the rotation angle and time when the microactuator is driven. f) Schematic diagram of the microactuator promoting the wound healing. g) The process of microactuators promoting the wound healing. h) Microactuator pulling hydrogels under the microscope.

FRSF‐based microactuators are constructed through over‐twisted yarn‐spinning techniques.^30^, ^31^ The FRSFs are initially twisted into the yarn by over‐twisting the fibers and then they are folded at the middle. Then one end of the yarn is released to remove the excrescent torque and form a self‐twisting structure (**Figure** **4**a). A torque‐balanced microactuator is formed, where the self‐twisting direction is the opposite of the initial twisting direction. In order to figure out the influence of the strand usage on the fabrication process and the torsional characteristics, a series of microactuators ranging from 2 to 10 ply fibers are made, where these microactuators are presented (Figure 4b). It is found that as the number of strands in microactuators varies from 2 to 10, the twist angle of two single yarns linearly increases from 15° to 38°.

### 2.5 Actuation Performance

The actuation behaviors driven by the water fog are evaluated through a self‐built apparatus that couple the tensile device with a high‐speed camera system (Figure 4c). During the tests, one end of the microactuator is fixed on a cantilever, while another end is loaded with a paddle, whose two sides are painted with blue and white colors (Figure 4d). The paddle weight is 59.8 mg with a value that is around 200 times larger than the weight of microactuators. A high‐speed micro‐camera system is employed to record the actuated rotation. The quantitative data are directly extracted from the recorded video through a frame‐by‐frame analysis.

Figure 4d,e presents the rotation of a 2 ply FRSF microactuator with the load. It is observed that the time difference between the water contact with the microactuator and the fiber torsion is less than 0.8 s, exhibiting an extraordinarily rapid response for the actuation of the water fog. Therefore, the projection width variation of the paddle is calculated to measure the degree of the rotation. It should be indicated that blue and white colors in each side of the paddle provide a convenient identity of the rotation. The rotation degree can be calculated in the form below

(1) $$\theta \left(t\right)=\arccos\;\frac{d\left(t\right)}{d_{0}}$$

where *d*(*t*) and *d*_0_ are the projection width of the paddle at time *t* and the corresponding width of the paddle, respectively. By calculating the angle variations over time, the correlation between time and rotation angle is established. The microactuator is accelerated to its max‐rotation speed within 3.5 s, which exceeds 1488.5° s^−1^ (248 rotations per minute, rpm) which means that the average rotation speed is 425.3° s^−1^ (Figure 4e and Figure S6, Supporting Information). It is found that the maximum angular acceleration is 739.9° s^−2^ (12.9 rad s^−2^). As a result, the twisting moment that the microactuator can provide approaches to 0.11 Nm kg^−1^, which is 1.7 times higher than that of the *B. mori* CSF actuators with the same structure.^32^ In fact, it is even comparable with that of the graphene hydrogel fiber actuators.^33^ FRSF microactuators show perfect reversible torsional deformations during cyclic processes. Furthermore, total actuation power of the FRSF microactuator can be programmed, because it is directly decided by the numbers of the used FRSFs. For example, an FRSF microactuator constructed by two single fibers can generate actuation power of 0.77 W kg^−1^. As the number of FRSFs increases from 2 to 10, the acceleration time, maximum speed, total duration, and the actuation power increase from 3.4 s, 248 rpm, 11.3 s, and 0.77 W kg^−1^ to 4.9 s, 1030 rpm, 38.9 s, and 2.1 W kg^−1^, respectively (Figure S7, Supporting Information).

This outstanding actuation performance of FRSF microactuators converts them as ideal choices for numerous applications, including the artificial muscle, linear actuator for locks, smart fabrics, humidity sensors, and so on.^34^, ^35^ In the following section, it is intended to show how these FRSF microactuators can be designed to drive wound healing (see Figure 4f). As a prototype, an agar hydrogel was used to mimic the human skin. Then FRSF microactuators are fixed at both ends of two agar gels separated with a gap of 2 mm. Furthermore, water fog is sprayed from the FRSF microactuator side. In such a process, the water fog can trigger the torsion of FRSF microactuators and contract the overall length, thereby driving the hydrogels to get close to each other (Figure 4g and Movie S2, Supporting Information). Microscopic images clearly show the process, where the screw pitch decreases as the number of spirals increases, thereby shortening the microactuator as a whole (Figure 4h and Movie S3, Supporting Information).

## 3 Discussion

### 3.1 Actuation Mechanism of the FRSF Microactuator

**Figure 5:**
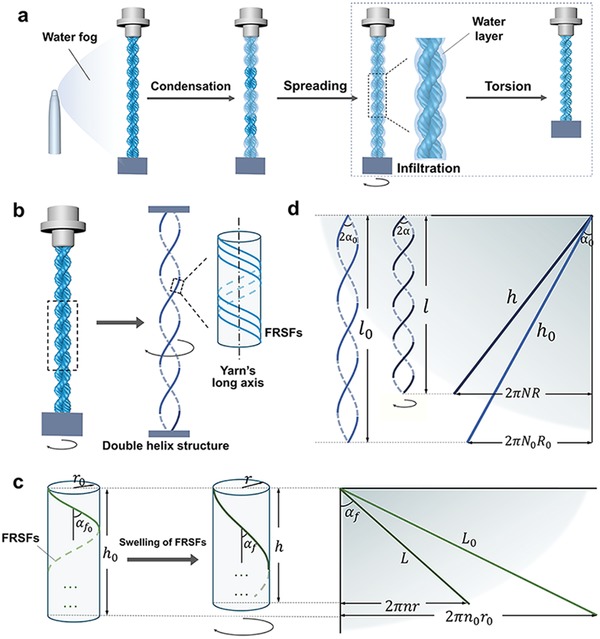
The mechanism of the water‐fog driven microactuators. a) Schematic diagram of the process of the water‐fog‐motivated microactuator. b) Two parts of the FRSF microactuator. c) The process of despiralization after the water infiltration and the FRSF swelling. d) The process of increasing the number of helices by rotation of the double helix structure.

In order to determine the actuation mechanism of the FRSF microactuator, a high‐speed micro‐camera system is employed to monitor the whole actuation process. As illustrated in **Figure** **5**a and Figure S8a (Supporting Information), the water fog is initially condensed into small water droplets on the surface of the FRSF strands, which mainly consist of the hydrophilic sericin. In each unit structure of the microactuator, tiny water droplets located at the smaller diameter of the microactuator automatically move to the position with the larger diameter. During such a condensation process, the droplet sizes increase continuously. When the furthest droplet moves to the thickest end, the droplet infiltrates the whole microactuator.

The FRSF microactuator can be simplified into a double helix structure (Figure 5b). This structure is formed by two coiled FRSF yarns, while each yarn consists of *K‐*FRSFs where *K* is the number of the FRSF per yarn. In a single yarn, FRSFs are twisted along the yarn's long axis with a hand of spiral opposite to the yarn's twisting direction. In other words, if the yarn is the left‐handed helix, FRSFs in the yarn must be right‐handed helix and vice versa. For this kind of configuration, if a torsional deformation of the microactuator happens, the synergistic changes of the geometric structure of the FRSFs, single yarns, and the microactuator can be expressed as^36^, ^37^

(2) $$\delta n\approx -\delta r+\frac{\cos\alpha_{f_{0}}\left(1-\cos\alpha_{f_{0}}\right)}{{\text{sin}}^{2}\alpha_{f}\cos\alpha_{f}}\;\delta L$$

where *δn*, *δr*, *δL* denote the relative change rates of the pitch number of the single yarn, radius of the single yarn, and the FRSF length, respectively. α_*f*_ and $\alpha_{f_{0}}$ are the twist angle of the single yarn and the corresponding initial twist angle, respectively. It should be indicated that the twist angle is the angle between the FRSF and the fiber axis of the single yarn. Therefore, when FRSFs are swelled and contracted, their long axis will be shortened so that *δL* < 0, the corresponding radius will be increased, i.e., *δr* > 0 and subsequently it is found that *δn* < 0. These variations are schematically presented in Figure 5c. These expressions indicate that the swelling and contraction trigger untwisting of FRSFs in the single yarn. The swelling and contraction of animal silks have been widely studied. If unrestricted animal silks are contacted with polar solvent or exposed to solvent vapor, they can contract significantly along the long axis and swell along the radial direction.^38^ The length of fiber can be shortened 5–50%, depending on the species of silks. In terms of FRSFs, their contraction rate can reach 5–10%.^9^

For the FRSF microactuator, both ends are fixed (Figure 5d), thus the whole pitch number per unit length of the microactuator μ is also fixed.^36^, ^37^, ^39^ In other words, μ is a positive constant. Therefore, the untwisting of FRSFs in the single yarn leads to an imbalance in the overall structure of the microactuator due to the mutual restriction between the pitch number of single yarn (*n*) and the pitch number of the microacuator (*N*). This mutual restriction can be mathematically described as

(3) $$\frac{2\pi n}{L}\;+\;\frac{2\pi N}{h}=\;\mu \left(\text{a}\;\text{constant}\right)$$

Equation (3) indicates that as *n* decreases, *N* must increase to compensate for the decrease of the first term. In other words, the untwisting of FRSFs in the single yarn that is triggered by the swelling and the contraction of FRSFs leads to the spontaneous rotating and twisting of the microactuator to increase the corresponding pitch numbers. However, with the progress of the water infiltration, the amount of water inhaled in microactuator is going to be saturated; the swelling and contraction of FRSFs also will reach equilibrium. Resultantly, after a period of rotation, the rotation speed of the microactuator gradually slows down until it stops.

### 3.2 Influence of the Rough and the Gradient Surface on the Actuation Rate

One of the unique characteristics of the FRSF microactuator compared to other microactuators is the simultaneous presence of the rough and the gradient surface. Therefore, it is intended to investigate the influence of this surface feature on the response rate of microactuators, which is hereafter called the infiltration rate. The larger droplets are more likely to cluster on the external side of the FRSF microactuator during the infiltration (Figure S8b, Supporting Information). It should be indicated that the external side area has the largest radius in the vertical projection direction. This indicates that the water droplets transport directionally on the microactuator surface. This droplet characteristic can be interpreted through the combination of the Wenzel equation^40^, ^41^ and the Laplace pressure difference.^41^, ^42^, ^43^ According to the Wenzel equation,^40^, ^41^ the surface gradient can lead to the wettability gradient, thereby driving the droplet movement to a place with higher roughness. Such a driving force can be expressed as^41^, ^44^, ^45^

(4) $$F_{\text{roughness}}=\int_{l_{1}}^{l_{2}}\gamma \left(\cos\theta_{\text{A}}-\cos\theta_{\text{R}}\right)\text{d}l$$

where θ_A_ and θ_R_ are the advancing and receding angles of the water drop on the microactuator, respectively. γ and d*l* denote the surface tension of the water drop and the movement distance of the water drop, respectively. *l*_1_ and *l*_2_ indicate the inner and external positions of the micro‐drive unit structure, respectively.

**Figure 6:**
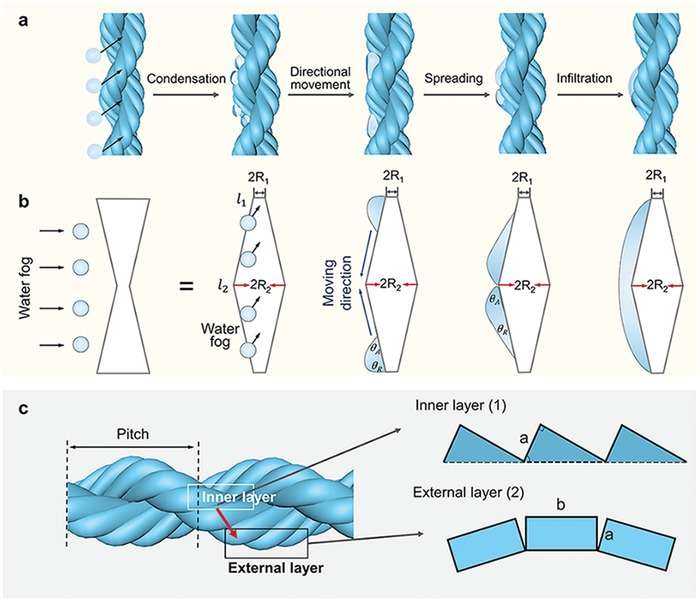
Influence of the microactuator structure on the driving droplet. a) The motion of the water droplets on the microactuator surface. b) Schematic diagram of water directional movement on a shape gradient surface. c) Structure of the internal and external layers of microactuators. The red arrow indicates the movement direction of the water drop.

However, the Laplace pressure difference typically causes the droplet to have a higher pressure at the slender side. Thus, the internal imbalance in the pressure forces the droplet to move from the inner position to the external position, where the movement path is presented in **Figure** **6**a,b. This can be mathematically described as^41^, ^42^, ^43^

(5) $$\Delta P_{\text{curvature}}=\;-\int_{R_{1}}^{R_{2}}\frac{2\gamma }{{\left(R+R_{0}\right)}^{2}}\;\sin\alpha \;\text{d}R$$

where *R*_1_ and *R*_2_ are the radii of the inner and external parts of the microactuator, respectively, when the microactuator is approximated by a cylinder. *R*_0_ is the radius of the water droplet.

The theoretical contributions of the surface roughness gradient on the infiltration rate of microactuators can be roughly estimated based on the subtraction of the spreading time of microactuators with rough surface *t*_s_ and the spreading time of microactuators with smooth surface *t*_r_ as

(6) $$\Delta t\;=t_{\text{r}}\;-\;t_{\text{s}}=\frac{\sqrt{\varepsilon }}{\sqrt{\varphi +\delta }}\;-\;\frac{\sqrt{\varepsilon }}{\sqrt{\varphi }}=\frac{\sqrt{\varepsilon }\left(\sqrt{\varphi }-\sqrt{\varphi +\delta }\right)}{\sqrt{\varphi \left(\varphi +\delta \right)}}$$

where φ = 6*γπNR*_0_^2^sin α, ε = 8*πρR*_0_^3^ *lN* ^2^(*R*_2_ − *R*_1_ + *R*_0_), and $\delta \;=\;3\gamma \left[\frac{2a}{b}+1-\sqrt{2}\sin\left(\alpha_{f}+\frac{\pi }{4}\right)\right]l(R_{2}-R_{1}+R_{0})\cos\theta$. φ and ε are parameters, which only depend on the shape gradient. δ is positively correlated with the roughness gradient so Δ*t* is always smaller than 0. This equation revealed that the surface roughness gradient caused by this spiral structure can substantially accelerate the infiltration of water droplets. It means that the rough and the gradient surface enable the rapid response of the microactuator to the water fog. Of note, for the sake of simplification, in this derivation (Appendix 2, Supporting Information) we only consider the spreading time. Therefore, Equation (6) only describes the motion of the drops on the external part of the yarn instead of the entire infiltration process. The water infiltration also includes a process that water infiltrates into the internal yarn structure, whose dynamics are given by Washburn's law of capillary imbibition, with a strong role of viscosity in the microstructure.

## 4 Conclusion

Programmable silk fiber microactuators can be achieved by combining the force‐reeling and the yarn‐spinning technique. Force‐reeling technique allows harvesting of silkworm silk fibers with structural uniformity and mechanical performance that are comparable with spider dragline silks. On the other hand, the yarn‐spinning technique is applied to construct microactuators with sophisticated helical structures. In this way, humidity‐induced microactuators were developed. In the obtained FRSF microactuator, the directional transports generated by the surface gradient, substantially promote the infiltration of water through the microactuator, thereby accelerating the microactuator response on the water fog. They exhibit a maximum rotation speed of 1030 rpm, a value that is bigger than humidity‐driven microactuators made by cotton, flax wool, or Tencel^32^ and even comparable with the CNT microactuators operated by electricity.^2^ The torsional deformation of the helical configuration, on the other hand, channels the actuating power of the silk fiber microactuators. A quantitative correlation between the helical configuration and the actuating power is derived in the present study so that the required actuating power for different applications can be programmed. Benefiting from superiorities of silk fiber microactuators in high mechanical performance, versatile processability, and excellent biocompatibility, they are expected to be applied in multiple emerging fields, such as biomedicine, smart textiles, soft robotics, and human–machine interfaces.

## 5 Experimental Section

*Force‐Reeling of A. Pernyi Silk Fibers*: *Antheraea pernyi* silkworms were obtained from the wild oak forest in Dandong, Liaoning province, China. When silkworms start spinning, the heads of the silkworms were fixed to prevent it from swinging from side to side. The silk fibers were directly pulled out from the mouthpart of the silkworms with reeling speeds from 5 mm s^−1^ to 30 mm s^−1^ and collected by a rotating cylinder. The morphology characterization of FRSFs and CSFs was carried out by using polarizing optical microscope (Olympus BX51‐P, Japan) and HRSEM (JEOL JSM‐7800F, Tokyo, Japan) at an acceleration voltage of 5 kV. All the samples were coated with a gold layer with a spray time of 20 s to provide conductivity before observation.

*Mechanical Testing*: A 50 mm FRSF was cut into three segments with lengths of 10, 25, and 10 mm, respectively. Two 10 mm FRSF segments were employed to measure the cross‐sectional areas of the FRSF, and the 25 mm FRSF segment was used for the tensile test. In a typical sample preparation process, a 25 mm FRSF was mounted on a cardboard frame with a base length of 20 mm and fixed with cyanoacrylate. After drying cyanoacrylate overnight, the frame was mounted in the testing machine (Instron 5966 machine, Instron, Norwood, USA) and the side supports of the frame were cut away so that the force was transmitted through the FRSF. Meanwhile, the initial length of the testing FRSF was measured at zero load point—a position in which the FRSF was tight but no force exerted on it. Further, a constant tensile speed (2 mm min^−1^) was applied to the FRSFs until they broke. All the mechanical tests were carried at 20–25 °C with the relative humidity of ≈43%. To calibrate the fibers' cross‐sectional area, the reserved two FRSF segments were aligned parallelly on the cardboard with a rectangle hollow in the middle, then the cardboards were covered with polydimethylsiloxane. After vacuuming to remove the air bubbles and curing for 3 h in the drying oven at 60 °C, the cross‐sectional areas of fibers were observed with SEM and were estimated with ImageJ software. The average value obtained from two segments was used as the cross‐sectional area of the testing sample for the calculation of its tensile stress.

*S‐FTIR Microspectroscopy of Single FRSF*: The experiments were performed at BL01B in the Shanghai Synchrotron Radiation Facility (SSRF), Shanghai, China. S‐FTIR spectra were recorded using a Nicolet 6700 FTIR microspectroscopy. The spot size (aperture) in all experiments was fixed as 10 × 10 µm^2^. Such a square aperture, on the one hand, guaranteed enough IR light to irradiate on the sample, on the other hand, avoided light diffraction that caused by size effect of FRSF, since the size of 10 × 10 µm^2^ was smaller than the dimension of single FRSF. The spectra were collected in the mid‐infrared range of 800–3800 cm^−1^ at a resolution of 4 cm^−1^ with 256 coadded scans. In order to characterize the secondary structural information of FRSF, deconvolution of amide III bands was carried out using PeakFit 4.12.^16^, ^29^ The number of peaks and their positions were obtained from the second derivative spectra and fixed during the subsequent deconvolution process.

*X‐Ray Diffraction Experiments*: WAXS and SAXS were performed to investigate the mesostructures of single FRSF. WAXS experiments were carried out at Characterization and Analysis Center of ShanghaiTech University by using Xenocs WAXS equipment, Xeuss 2.0. The diffraction patterns were collected by the detector with 172 pixels × 172 pixels of 172 µm × 172 µm area each. The wavelength and the photon flux of the X‐ray source was 1.54189 Å and 4.0 × 10^7^ photons s^−1^, respectively. The beam size at the detector was 1.2 mm × 1.2 mm. SAXS experiments were carried out in Shanghai synchrotron source beamline BL19U2, Shanghai, China, with a wavelength of 1.03 Å, delivering a high‐photon flux (5 × 10^12^ photons s^−1^) onto the sample. A complementary metal‐oxide‐semiconductor (CMOS) hybrid pixel detector, with a total number of 172 pixels × 172 pixels, was employed to collect the diffraction patterns. The beam size at the detector was fixed as 0.33 mm (horizontal) × 0.05 mm (vertical).

*Preparation of FRSF Microactuators*: FRSF‐based microactuators were constructed through over‐twisted yarn‐spinning techniques. The FRSFs were first tied between an electric motor and a movable load which was initially 40 cm away and then they were twisted into the yarn by over‐twisting the fiber with the speed of 7500° min^−1^ for 5 min. They were folded at the middle immediately after the over‐twisting process. Then one end of the yarn was released to remove the excrescent torque and form a self‐twisting structure. Then a torque‐balanced microactuator was formed, where the self‐twisting direction was the opposite of the initial twisting direction. In order to figure out the influence of the strand usage on the fabrication process and the torsional characteristics, a series of microactuators ranging from 2 to 10 ply fibers was made.

*Actuation Performance Evaluation*: The actuation behavior that driven by water fog was evaluated through a self‐built apparatus that coupled a tensile deceive with a high‐speed camera system. During the tests, one side of the microactuator was fixed on a cantilever, and another side was loaded with a paddle (59.8 mg in weight), which was painted with blue and white color, respectively. A humidifier with a constant power was fixed in the apparatus to generate water fog for humidification of the suspended part of the FRSF microactuators. The whole actuation processing was monitored by using a telephoto lens‐based high‐speed camera system (i‐SPEED 716, iX Camera, UK). The quantitative time‐torsion angle relationship was obtained through frame‐by‐frame analysis of the recorded movies.

*Derivation of the Driving Mechanism of FRSF Microactuator*: Two relations between the length of single FRSF in yarn (*L*) and the length of the twisted yarn (*h*) were obtained from the geometrical changes shown in Figure 5c. They followed that

(7) $$L^{2}=h^{2}\;+{\left(2\pi nr\right)}^{2}$$

(8) $$2\pi nr\;=\;L\;\sin\alpha_{f}=\;h\tan\alpha_{f}$$

where *n* is the pitch number of the single yarn, *L* is the length of FRSF, *h* is the length of single yarn, *r* is the radius of the single yarn, α_*f*_ is the twist angle of the single yarn.

By taking the second derivative of *L* from both sides of Equation (7), Equation (9) was obtained

(9) $$LdL\;=\;hdh+4\pi^{2}r^{2}ndn+4\pi^{2}n^{2}rdr$$

Substituting Equation (8) into Equation (9), Equation (10) was obtained

(10) $$\frac{\Delta n}{n}=\frac{\Delta L}{L{\text{sin}}^{2}\alpha_{f}}\;-\;\frac{\Delta h}{h{\text{tan}}^{2}\alpha_{f}}\;-\;\frac{\Delta r}{r}$$

The geometrical relation between ∆*h* and ∆*L* is illustrated in Figure S9 (Supporting Information). In such a geometrical relation, sin $\alpha_{f}=\;\frac{2\pi nr}{L}$. For FRSF microactuators, the radius of a single yarn was around 50 µm and the change in the twisting turns due to torsion deformation was generally smaller than 50 turns. Therefore, when the length of the single yarn was long enough, the change between twisting angle before and after torsion, i.e., $\alpha_{f_{0}}-\alpha_{f}$, was very small. Taking this case as an example, the length of single yarn was 45 cm, which was around 9000 times larger than the radius of the single yarn, thus the angle of $\alpha_{f_{0}}-\alpha_{f}$ was smaller than 10^−3^ rad. As a result, in triangle ABC (Figure S9, Supporting Information), angle β and γ were both approximately equal to 90°. Based on this approximation, together with the geometrical relation as shown in Figure 5c and Figure S9 (Supporting Information), the following equations were obtained

(11) $$h\;=\;L\cos\alpha_{f}$$

and

(12) $$\Delta h\;=\left(\Delta L\cot\alpha_{f_{0}}-\;L\sin\Delta \alpha_{f}\right)\sin\alpha_{f_{0}}$$

Substituting these two equations into Equation (10), the following equation was obtained

(13) $$\frac{\Delta n}{n}=\;-\frac{\Delta r}{r}+\frac{\cos\alpha_{f_{0}}\left(1-\cos\alpha_{f_{0}}\right)}{{\text{sin}}^{2}\alpha_{f}\cos\alpha_{f}}\;\frac{\Delta L}{L}+\frac{\sin(\alpha_{f_{0}}-\alpha_{f})\sin\alpha_{f_{0}}\cos\alpha_{f}}{{\text{sin}}^{2}\alpha_{f}}$$

Since $\alpha_{f_{0}}-\alpha_{f}\approx 0,$ Equation (13) was reduced to

(14) $$\delta n\approx -\delta r+\frac{\cos\alpha_{f_{0}}\left(1-\cos\alpha_{f_{0}}\right)}{{\text{sin}}^{2}\alpha_{f}\cos\alpha_{f}}\delta L$$

where *δn*, *δr*, *δL* are relative change rate of pitch number of single yarn, the radius of single yarn, and FRSF length, respectively.

## Conflict of Interest

The authors declare no conflict of interest.

## Supporting information

Supporting Information

Supplemental Movie 1

Supplemental Movie 2

Supplemental Movie 3
