# Supplementary material for: Ultrastrong and Highly Sensitive Fiber Microactuators Constructed by Force‐Reeled Silks
Source: Adv Sci (Weinh). 2020 Jan 16;7(6):1902743. doi: 10.1002/advs.201902743 (PMC7080530; doi:10.1002/advs.201902743)
Supplement: Supplementary file 1 — Supporting Information [file ADVS-7-1902743-s001.pdf]

Copyright WILEY-VCH Verlag GmbH & Co. KGaA, 69469 Weinheim, Germany, 2020.

ADVANCED SCIENCE Open Access

Supporting Information

for *Adv. Sci.,* DOI: 10.1002/advs.201902743

Ultrastrong and Highly Sensitive Fiber Microactuators Constructed by Force-Reeled Silks

*Shihui Lin, Zhen Wang, Xinyan Chen, Jing Ren, and Shengjie Ling**

Copyright WILEY-VCH Verlag GmbH & Co. KGaA, 69469 Weinheim, Germany, 2020.

Supporting Information

Ultrastrong and Highly Sensitive Fiber Microactuators Constructed by Force-Reeled Silks

*Shihui Lin, Zhen Wang, Xinyan Chen, Jing Ren, and Shengjie Ling**

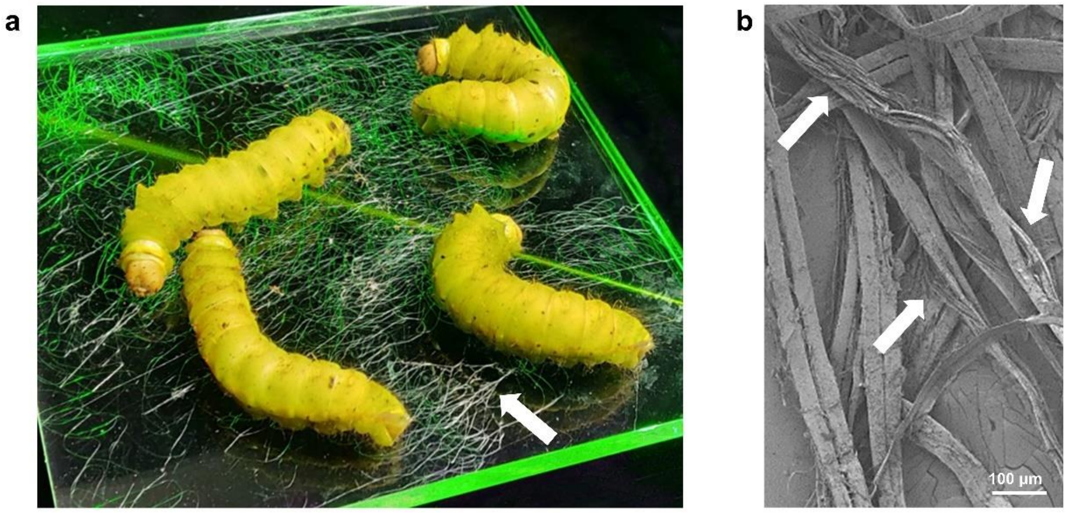

Figure S1. Defects in the natural spinning process. (a) Trajectory of *A. pernyi* silkworms spinning on the acrylic plane. (b) SEM image of silk fibers silkworms spun on the plane. The arrow indicates the defects in the silk fibers caused by the motion of the silkworms' mouthparts during spinning.

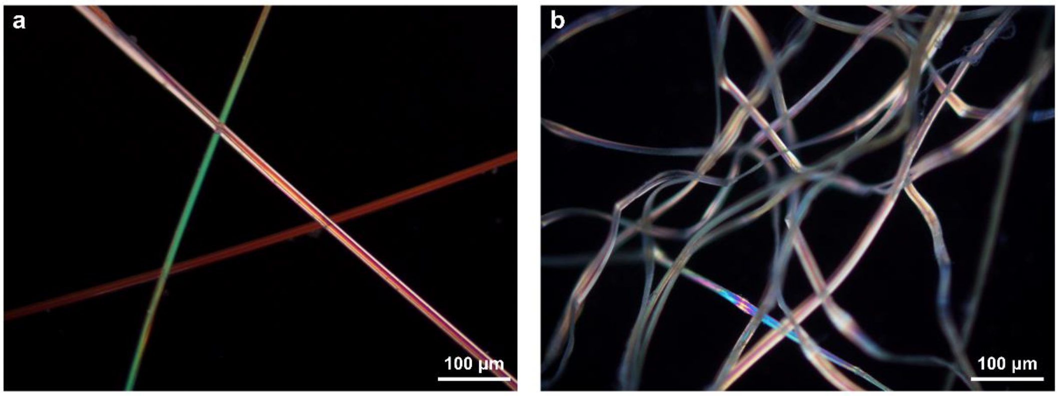

Figure S2. Images of force reeled silk fibers (a) and cocoon silk fibers (b) under polarized light microscope.

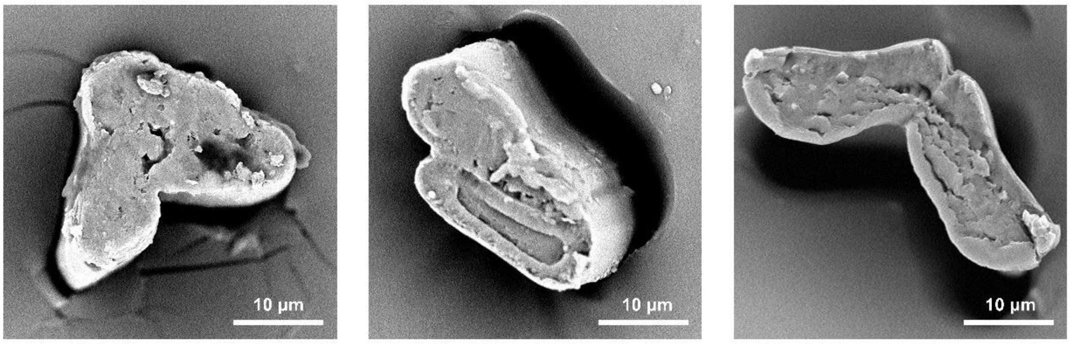

Figure S3. SEM images of the cross sections of FRSFs.

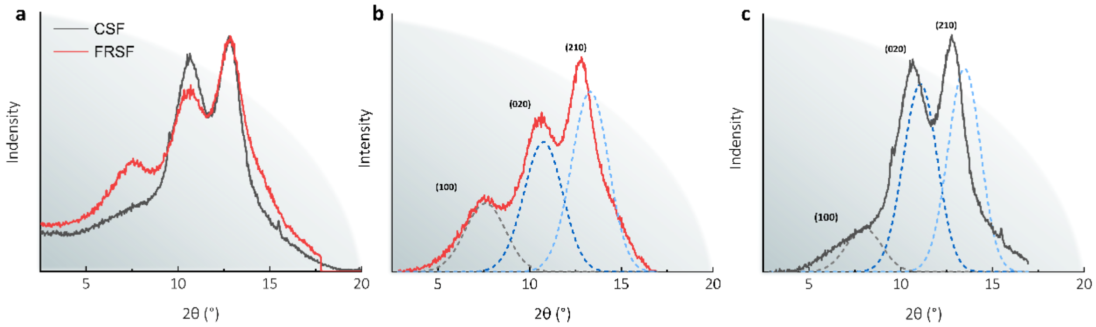

Figure S4. The equatorial intensity distribution curve from two-dimensional WAXS image. (a) Equatorial intensity distribution of CSF and FRSF. (b) Peak fitting of FESF. (c) Peak fitting of CSF.

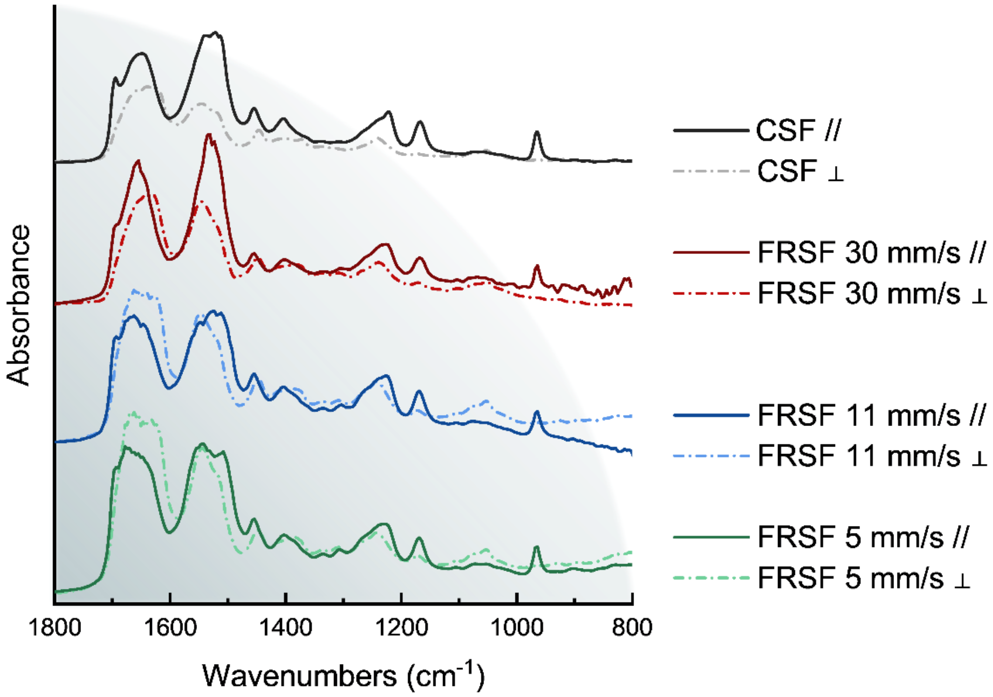

Figure S5. FTIR spectra of CSF and FRSFs at different reeling speeds.

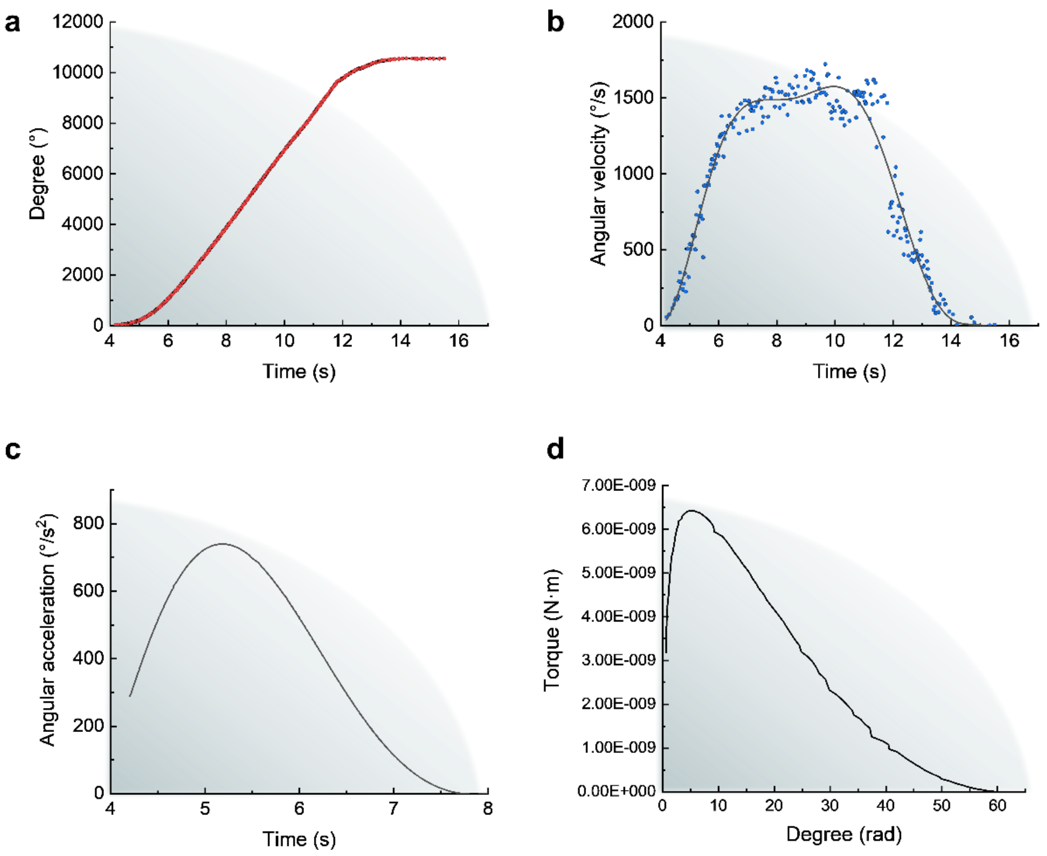

Figure S6. Actuation performance of 2-ply microactuator. (a) The curve of the degree of rotation angle and time. (b) The curve of the angular velocity and time. The blue points are derived from the time derivative of data in (a), and the black line is the curve obtained by polynomial fitting of the scattered blue points. (c) The curve of the angular acceleration and time. Due to the large dispersion of data, the angular acceleration-time curve is obtained by taking the derivative with respect to time of the previous angular velocity-time fitting curve. (d) The curve of the torque and degree. The torque is further derived from the fitting curve of angular acceleration in (c).

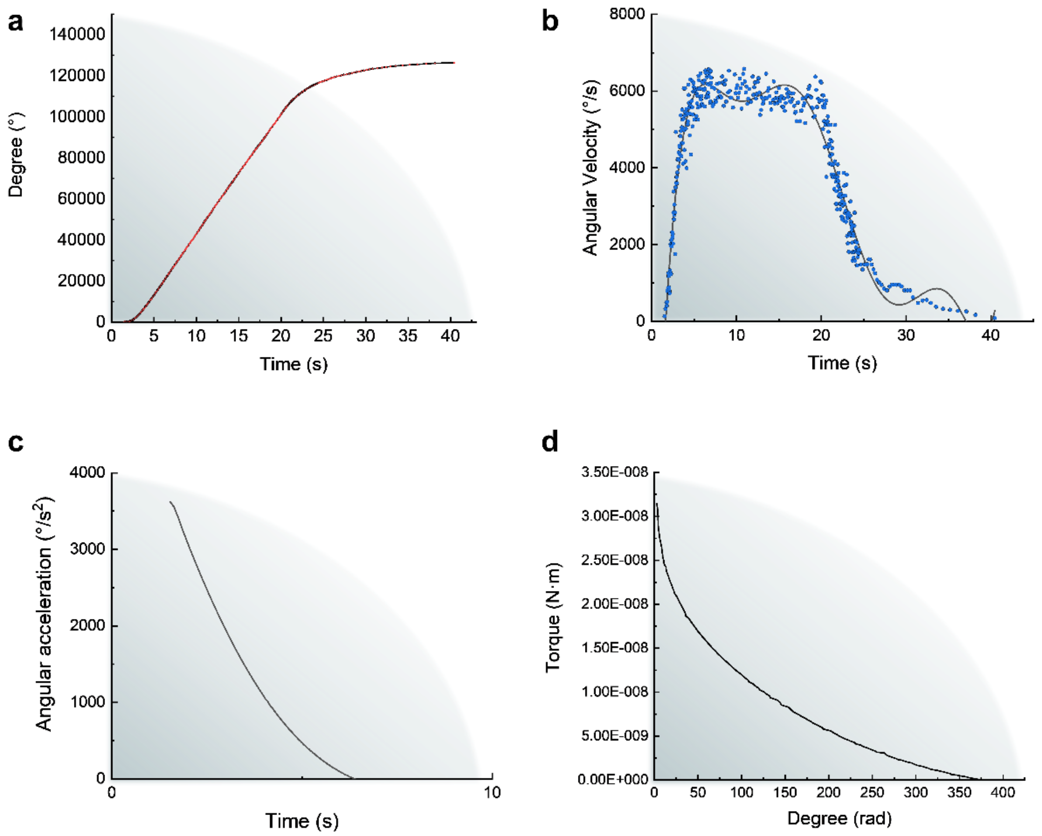

Figure S7. Actuation performance of 10-ply microactuator. (a) The curve of the degree of rotation angle and time. (b) The curve of the angular velocity and time. (c) The curve of the angular acceleration and time. Due to the large dispersion of data, the angular acceleration-time curve is obtained by taking the derivative with respect to time of the previous angular velocity-time fitting curve. (d) The curve of the torque and degree. The torque is further derived from the fitting curve of angular acceleration in (c).

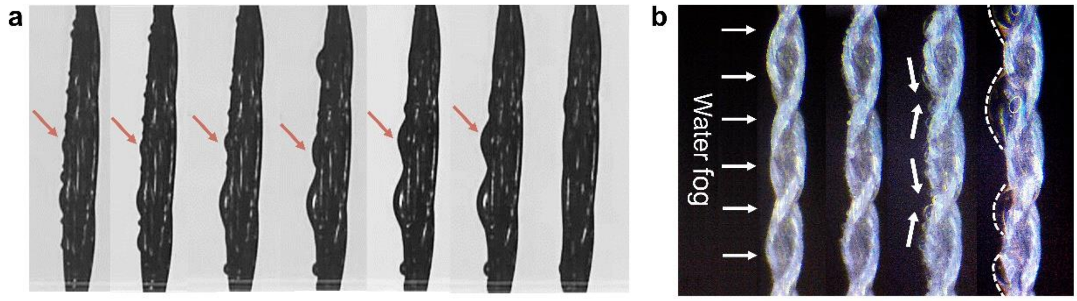

Figure S8. The madefaction process of microactuators. (a) The movement of water droplets recorded with high-speed camera. The red arrow indicates the position of the water droplets. (b) The movement of water droplets under a microscope. The white dotted line highlights the position of the water droplets.

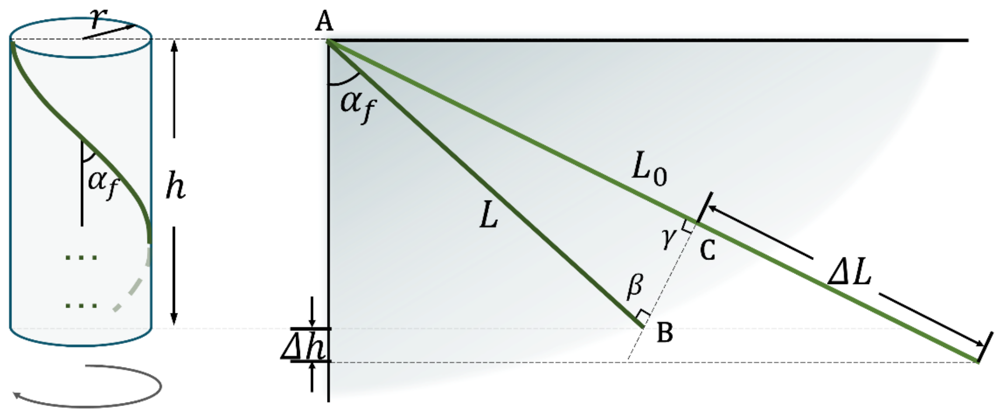

Figure S9. The geometrical relation between Δh and ΔL.

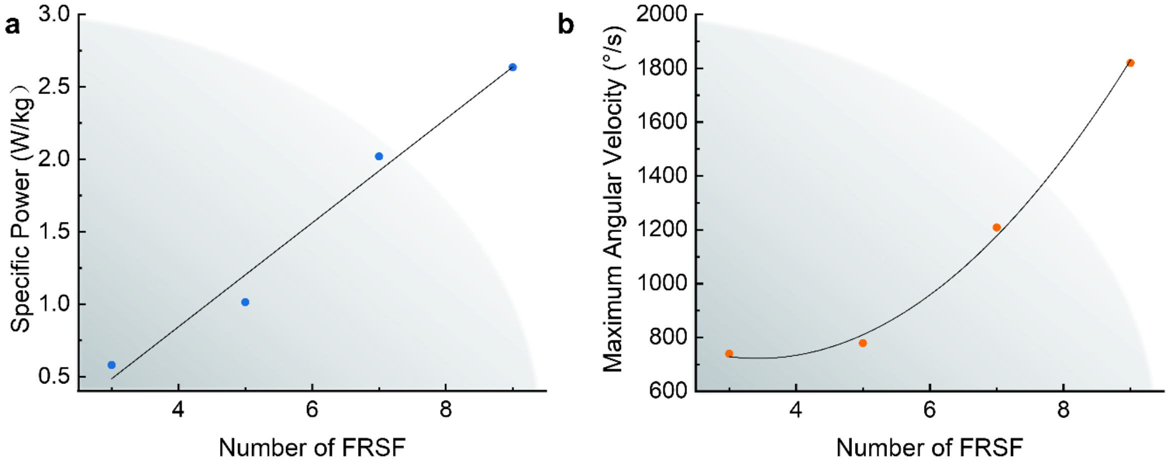

Figure S10. (a) The relationship of the specific power and the number of FRSF. (b) The relationship of the maximum angular velocity and the number of FRSF. These tested microactuators were prepared under the same conditions except for the number of FRSF.

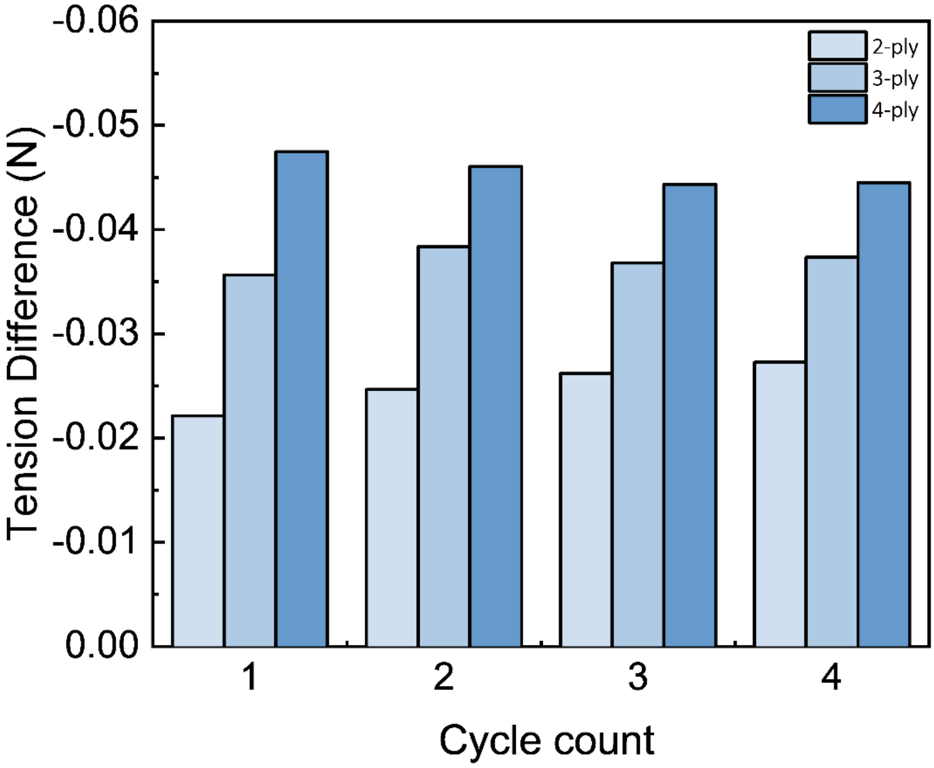

Figure S11. Mechanical difference of microactuators before and after water infiltration.

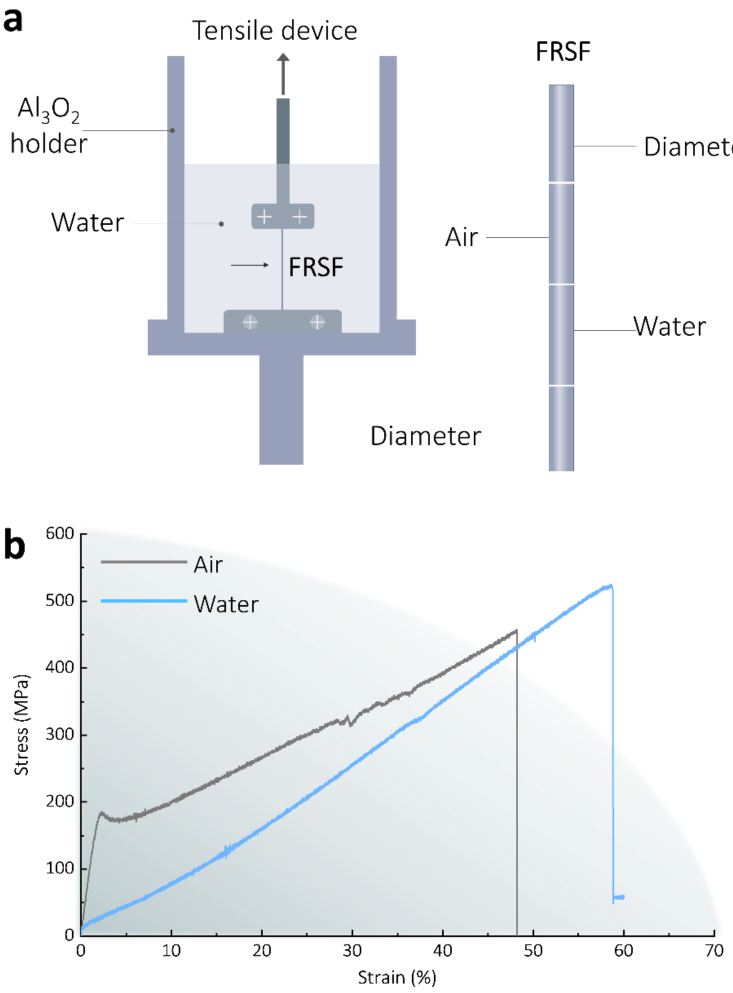

Figure S12. Mechanical properties of FRSF in water. (a) The structure of the selfmade sample pool. (b) The stress- strain crave of FRSF in water and in air.

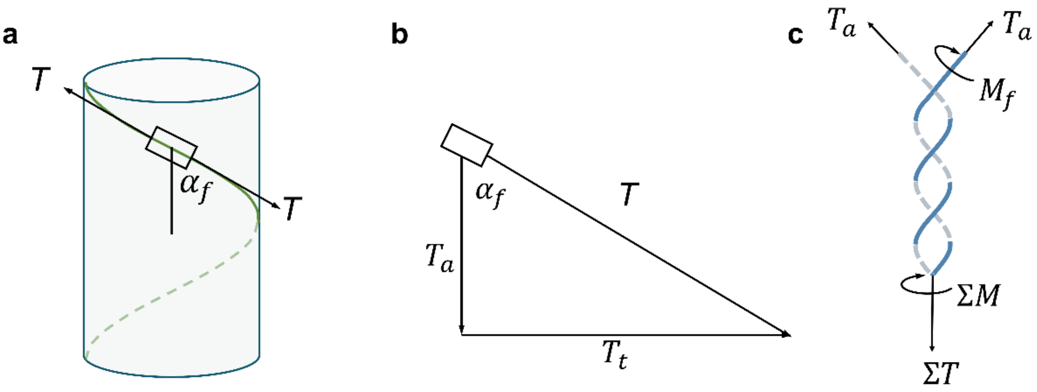

Figure S13. Force analysis of microactuator.

Table S1. The specific values of the density, strength, Yang's modulus and the toughness of the FRSF and different natural and synthetic fibers.

	Density (g cm^{-1})	Strength (MPa)	Strain (%)	Modulus (Gpa)	Toughness (MJ m^{-3})
N. clavipes dragline silk	1.3	925±45	17.5±0.5	12±1	110
Kevlar 49 fiber	1.44	3600	2.7	130	50
High-tensile steel	7.82	1650	0.8	200±10	6
FRSF	1.3	571±97	31±6	11±2	131±77
CSF	1.3	359±83	24±8	7±2	61±28

Table S2. Actuation performance of 2-ply microactuators prepared with different silk materials.

Material of microactuator	Maximum speed (rpm)	Average speed (rpm)
CSF	51	34
PSF	116	71
FRSF	248	115

Appendix 1. Calculation of microactuator working parameters

For 2-ply microactuator, the average rotation speed was calculated from the curve of the degree of rotation angle and time in Figure S6a :

$$\bar{\omega} = 932.5\,°\,s^{-1} = 155\,rpm \tag{S1}$$

And the relations of the degree of angle (θ), angular velocity (ω) and angular acceleration (α) are:

$$\omega = \frac{d\theta}{dt} \tag{S2}$$

$$\alpha = \frac{d\omega}{dt} \tag{S3}$$

As shown in Figure S6b, we took the time derivative of the degree of rotation angle to figure out the angular velocity. We then obtained ω-t curve of the propeller by polynomial fitting. In this process, the "bad points" were removed to ensure the valid standard deviation of the fitting. Thereafter, the α-t curve can be obtained by taking the derivative with respect to time of the previous ω-t fitting curve (Figure S6c). When the $\alpha = 0$, rotation speed (ω) reaches its maximum, so we can know from Figure S6b and Figure S6c that the microactuator accelerated to its maximum rotation speed from 4.3 s to 7.7 s.

$$\omega_{max} = 1488.5\,°\,s^{-1} = 248\text{ rpm}$$

The mass of the microactuator was:

$$m_{microactuator} = 6.0 \times 10^{-5} g \tag{S4}$$

For the paddle, the mass and moment of inertia were:

$$m_{paddle} = 5.98 \times 10^{-5}\,kg \tag{S5}$$

$$I = \frac{1}{12} m_{paddle} {l_{paddle}}^2 = 4.98 \times 10^{-10}\,kg \cdot m^2 \tag{S6}$$

The work done by the microactuator on the paddle could be integrated by the torque-angle curve,

$$W = \int M d\theta = \int I\alpha d\theta = 1.6 \times 10^{-7}\ \text{J} \qquad \text{(S7)}$$

The work microactuator does on the paddle make it accelerate. The average actuation power is the work microactuator dose divided by the duration of the acceleration.

$$\bar{P} = \frac{W}{t} = \frac{1.6\times10^{-7}\ \text{J}}{3.5\ \text{s}} = 4.6 \times 10^{-8}\ \text{W} \qquad \text{(S8)}$$

$$P_{specific} = \frac{\bar{P}}{m_{microactuator}} = 0.77\ \text{W/kg} \qquad \text{(S9)}$$

The $\bar{\omega}$, ω_{max}, $P_{specific}$ of the 10-ply microactuator (Figure S7) also can be calculated by following the same processing, the results listed as follows

$$\bar{\omega} = 3113.6\ {}^{\circ}\, s^{-1} = 519\ \text{rpm} \qquad \text{(S10)}$$

$$\omega_{max} = 6179.3\ {}^{\circ}\, s^{-1} = 1030\ \text{rpm} \qquad \text{(S11)}$$

$$P_{specific} = \frac{\bar{P}}{m_{microactuator}} = 2.1\ \text{W/kg} \qquad \text{(S12)}$$

Appendix 2. Infiltration time influenced by roughness and the surface gradient

According to Wenzel equation, the relationship between surface roughness gradient and contact angle is

$$\cos\theta_A = r_2 \cdot \cos\theta \qquad \text{(S13)}$$

$$\cos\theta_R = r_1 \cdot \cos\theta \qquad \text{(S14)}$$

where θ is the intrinsic contact angle, θ_A and θ_R are the advancing and receding angles of water drop on the microactuator, $r_1 = \cos\alpha_f + \sin\alpha_f$ is the roughness of the inner layer, $r_2 = \frac{(2a+b)}{b}$ is the roughness of the external layer. Due to the presence of hydrophilic sericin on the surface of the FRSFs, the intrinsic contact angle will be less than 90°, namely, $\theta < 90°$. From

Figure 6c, we can find $r_2 > r_1$, therefore, $\cos\theta_A > \cos\theta_R$. In such case, the gradient of the surface-free energy originated from surface roughness differences can produce a driving force, $F_{roughness}$, driving the water droplets directional movement from less rough areas (l_1 in Figure 6b) to areas with high roughness (l_2 in Figure 6b) as described as follows

$$F_{roughness} = \int_{l_1}^{l_2} \gamma(\cos\theta_A - \cos\theta_R)\, dl \tag{S15}$$

Assuming that a FRSF microactuator with a length of l is composed of N twisted turns (namely the number of the unit structure is N), therefore,

$$l_2 - l_1 = \frac{l}{2N} \tag{S16}$$

Substituting Equation S14 and S16 into Equation S15, we obtain

$$F_{roughness} = \frac{\gamma l\left[\frac{2a}{b}+1-\sqrt{2}\sin\left(\alpha_f+\frac{\pi}{4}\right)\right]\cos\theta}{2N} \tag{S17}$$

Accordingly, the work generated by the $F_{roughness}$ can be written as

$$W_{roughness} = \int_{l_1}^{l_2} F\, dl = \frac{\gamma l^2\left[\frac{2a}{b}+1-\sqrt{2}\sin\left(\alpha_f+\frac{\pi}{4}\right)\right]\cos\theta}{4N^2} \tag{S18}$$

In addition to the gradient of the surface free energy, the shape gradient of FRSF microactuator also plays a role in controlling the directional movement of the water droplets. As shown in Figure 5b, each twisted turn (or unit structure) of microactuator can be approximately simplified into a conical object. This type of conical shape can generate a Laplace pressure difference ($\Delta P_{curvature}$) between the two opposite sides of the water droplet. In this case, $\Delta P_{curvature}$ and its corresponding work can be read as

$$\Delta P_{curvature} = -\int_{R_1}^{R_2} \frac{2\gamma}{(R+R_0)^2}\sin\alpha\ dR = \frac{2\gamma\sin\alpha}{R_2-R_1+R_0} \tag{S19}$$

$$W_{curvature} = \int_{l_1}^{l_2} \Delta P_{curvature}\pi {R_0}^2\, dl = \frac{\gamma\pi {R_0}^2 l\sin\alpha}{N(R_2-R_1+R_0)} \tag{S20}$$

From Equation S18 and S20, together with two assumptions, that is, (i) The initial velocity of water droplet is zero and (ii) gravity has no significant effect on water droplets, we can directly deduce the time (t) required for water droplets to infiltrate microactuator (i.e., the

time for water droplets moving from l_1 to l_2) by using the kinetic energy theorem. In this case, the kinetic energy of a water drop can be written as

$$E_k = \frac{1}{2} m\Delta v^2 = W_{roughness} + W_{curvature} \tag{S21}$$

where Δv is the velocity of the water drop that produced by two driving forces; $m = \frac{4}{3}\pi\rho {R_0}^3$ is the mass of the water droplet. The average velocity ($\bar{v}$) of water droplets thus can be written approximately as

$$\bar{v} \approx \frac{1}{2}\Delta v = \sqrt{\frac{3\gamma l\left\{\left[\frac{2a}{b}+1-\sqrt{2}\sin\left(\alpha_f+\frac{\pi}{4}\right)\right]l(R_2-R_1+R_0)+4\pi N {R_0}^2 \sin\alpha\right\}}{32\pi\rho {R_0}^3 N^2 (R_2-R_1+R_0)}} \tag{S22}$$

Therefore, the time t_r for water droplets to infiltrate microactuator can be read as

$$t_r = \frac{l_2-l_1}{\bar{v}} = \sqrt{\frac{8\pi\rho {R_0}^3 l(R_2-R_1+R_0)}{3\gamma\left\{\left[\frac{2a}{b}+1-\sqrt{2}\sin\left(\alpha_f+\frac{\pi}{4}\right)\right]l(R_2-R_1+R_0)\cos\theta+4\pi N {R_0}^2 \sin\alpha\right\}}} \tag{S23}$$

Where ρ denote the density of water droplets, γ denote the surface tension of the water drop. R_1 and R_2 are the radii of the inner and external parts of the microactuator, respectively when the microactuator is approximated by a cylinder. R_0 is the radius of the water droplet. l and N are the length of the microactuator and the number of spirals in a microactuator. a and b are the length and width of the FRSF cross section, respectively. After plugging in these relevant parameters, the value of t_r can be calculated, $t_r \approx 1.5 \times 10^{-4}$ s.

For microactuators with a smooth surface (i.e., without grooves), only shape gradient exists, so in this case E_k, $\bar{v}_s$, and t_s can be expressed as follows

$$E_k = \frac{1}{2} m\Delta v^2 = W_{curvature} \tag{S24}$$

$$\bar{v}_s = \sqrt{\frac{3\gamma l\,\pi N {R_0}^2 \sin\alpha}{4\pi\rho {R_0}^3 N^2 (R_2-R_1+R_0)}} \tag{S25}$$

$$t_s = \sqrt{\frac{4\pi\rho {R_0}^3 l N(R_2-R_1+R_0)}{3\gamma\,\pi {R_0}^2 sin\,\alpha}} \tag{S26}$$

The theoretical contributions of the surface roughness gradient on the infiltration rate of microactuators can be roughly based on the subtraction of the infiltration time of microactuators with rough surface t_s and the infiltration time of microactuators with smooth surface t_r as:

$$\Delta t = t_r - t_s = \frac{\sqrt{\varepsilon}}{\sqrt{\varphi+\delta}} - \frac{\sqrt{\varepsilon}}{\sqrt{\varphi}} = \frac{\sqrt{\varepsilon}\left(\sqrt{\varphi}-\sqrt{\varphi+\delta}\right)}{\sqrt{\varphi(\varphi+\delta)}} \quad \text{(S27)}$$

Where $\varphi = 6\gamma\,\pi N {R_0}^2 sin\,\alpha$, $\varepsilon = 8\pi\rho {R_0}^3 l N^2 (R_2 - R_1 + R_0)$, and $\delta = 3\gamma\left[\frac{2a}{b} + 1 - \sqrt{2}\,sin\left(\alpha_f + \frac{\pi}{4}\right)\right] l (R_2 - R_1 + R_0)\,cos\,\theta$.

It should be indicated that φ and ε are parameters, which only depend on the shape gradient. Meanwhile, δ is positively correlated with the roughness gradient so that as the roughness difference between the outside and the inside of microactuator increases, the parameter δ increases. The roughness of the external side is always larger than that of the internal side, so δ is always a positive parameter (Figure 6c). Subsequently, Δt is always smaller than 0, indicating that the surface roughness gradient caused by this spiral structure can substantially accelerate the infiltration of water droplets. When water infiltrates the whole FRSF microactuator, the actuation deformation of the microactuator occurs. Accordingly, the time for the water to infiltrate the entire microactuator is approximately equal to the response time of the microactuator. It means that the rough and the gradient surface enable the rapid response of the microactuator to the water fog.

Of note, as mentioned, this derivation is based on two assumptions, that is, the initial velocity of the drop is zero and the effect of gravity on water droplets is ignored. Thus, the work produced by those two driving forces is totally converted into the kinetic energy of the water droplets. These two assumptions are rational because the diffusion direction of water fog is perpendicular to the long axis direction of the FRSF microactuator, so the water droplets condensed on the surface of microactuator has almost no initial velocity along the axial

direction. In addition, when the droplets are small in volume, the gravity of the droplets is much lower than the surface tension force. Thus, the effect of gravity on water droplets can be ignored.

Appendix 3. Experimental strategies for recording the actuation forces of FRSF microactuators and mechanical properties of FRSFs in water.

In order to record the force that generated by the FRSF microactuators, mechanical test was used for the measurement of the mechanical properties of the FRSF yarn (Figure S11). The yarn was fixed on both ends. We recorded the load-time curve (which has no displacement) during the water fog went in and out. The water fog was given by an ultrasonic humidifier which was placed 25 cm away from the yarn. In such a distance, we confirmed that water impact force had no detectable influence on the load of the yarn. In addition, in these experiments, we recorded the loads of FRSF microactuators only when they reached a platform.

To understand the driving force of the deformation, we designed a tensile accessory to test the mechanical properties of these FRSFs in water (Figure S12a). The control sample tested in air, During the test, a full-dried fiber sample was first fixed on the tensile grips, which was covered by a sealed tank. Water was then pumped into the tank and ensure that the whole sample was immersed in solvent during the tensile test. The test was conducted after 20 mins that make sure the fiber has been fully wetted by the solvent. The stress-strain curve in Figure S12b shows that the mechanical behavior of FRSFs is significantly different in water compared with it in air. The modulus drops sharply from 10.5±0.6 GPa in the air to 0.5±0.1 GPa in water.

Appendix 4. Theoretical calculation of structural influence on actuation power.

We conducted this analysis from the single strand yarn in FRSF microactuator. From the geometric relationship shown in Figure S13a,b, we have

$$T_a = T\cos\alpha_f \tag{S28}$$

$$M_f = \frac{KrT_t}{2} \tag{S29}$$

Before the FRSF microactuator contacts with water fog, its mechanical parameters, including the sum of the tension of K FRSFs (T_0), the tension of a single yarn (T_{a_0}), the sum of the torques applied to K FRSFs (M_{f_0}) can be written as

$$T_0 = \frac{KabE(L_0 - L_{initial})}{L_{initial}} \tag{S30}$$

$$T_{a_0} = \frac{KabE(L_0 - L_{initial})\cos\alpha_{f0}}{L_{initial}} \tag{S31}$$

$$M_{f_0} = \frac{Kabr_0E(L_0 - L_{initial})\sin\alpha_{f0}}{2L_{initial}} \tag{S32}$$

After the microactuator infiltrated by water, its mechanical parameters can be written as

$$T = \frac{KabE_w(L - L_{initial})}{L_{initial}} \tag{S33}$$

$$T_a = \frac{KabE_w(L - L_{initial})\cos\alpha_f}{L_{initial}} \tag{S34}$$

$$M_f = \frac{Kabr_0E_w(L - L_{initial})\sin\alpha_{f0}}{2L_{initial}} \tag{S35}$$

According to its geometrical relations, we can further have

$$\Sigma T = 2T_a\cos\alpha \tag{S36}$$

$$\Sigma M = 2M_f\cos\alpha + \Sigma TR\tan\alpha \tag{S37}$$

So that we can obtain

$$\Delta T = 2Kab\frac{E_w(L-L_{initial})\cos\alpha_f\cos\alpha - E(L_0-L_{initial})\cos\alpha_{f_0}\cos\alpha}{L_{initial}} \tag{S38}$$

$$\Delta M =$$

$$Kab\frac{rE_w(L-L_{initial})\sin\alpha_f\cos\alpha+2RE_w(L-L_{initial})\cos\alpha_f\sin\alpha}{L_{initial}} -$$

$$Kab\frac{r_0E(L_0-L_{initial})\sin\alpha_{f_0}\cos\alpha+2R_0E(L_0-L_{initial})\cos\alpha_{f_0}\sin\alpha}{L_{initial}} \tag{S39}$$

where $L_{initial}$ Is the length of FRSFs before twisting, K is the number of the FRSFs per yarn, ΔT is the whole structure's tension change, and ΔM is the whole structure's torque change.

Appendix 5. Effect of the mechanical properties of silk fibers on the actuation performance of microactuators.

To understand the effect of the mechanical properties of silk fibers on sensitivity (rotation speed) of the microactuators, the rotation speeds of 2-ply microactuators that made by the CSFs and PSFs were measured, and their results are listed in Table S2. This table reveals that the rotation speeds (sensitivities) of silk fiber microactuators are positively correlated with the mechanical properties of the fibers that used.
